# Beyond the whole-mount phenotype: high-resolution imaging in fluorescence-based applications on zebrafish

**DOI:** 10.1242/bio.042374

**Published:** 2019-05-15

**Authors:** Veronika Oralová, Joana T. Rosa, Mieke Soenens, Jan Willem Bek, Andy Willaert, Paul Eckhard Witten, Ann Huysseune

**Affiliations:** 1 Evolutionary Developmental Biology, Biology Department, Ghent University, 9000 Ghent, Belgium; 2 Institute of Animal Physiology and Genetics, Czech Academy of Sciences, 602 00 Brno2, Czech Republic; 3 Center of Marine Sciences (CCMAR), University of Algarve, 8005-139 Faro, Portugal; 4 Center for Medical Genetics Ghent, Ghent University, 9000 Ghent, Belgium

**Keywords:** Zebrafish, GMA, GFP, Fluorophores, Immunofluorescence, Cell tracking, TRAP

## Abstract

Zebrafish is now widely used in biomedical research as a model for human diseases, but the relevance of the model depends on a rigorous analysis of the phenotypes obtained. Many zebrafish disease models, experimental techniques and manipulations take advantage of fluorescent reporter molecules. However, phenotypic analysis often does not go beyond establishing overall distribution patterns of the fluorophore in whole-mount embryos or using vibratome or paraffin sections with poor preservation of tissue architecture and limited resolution. Obtaining high-resolution data of fluorescent signals at the cellular level from internal structures mostly depends on the availability of expensive imaging technology. Here, we propose a new and easily applicable protocol for embedding and sectioning of zebrafish embryos using in-house prepared glycol methacrylate (GMA) plastic that is suited for preservation of fluorescent signals (including photoactivatable fluorophores) without the need for antibodies. Four main approaches are described, all involving imaging fluorescent signals on semithin (3 µm or less) sections. These include sectioning transgenic animals, whole-mount immunostained embryos, cell tracking, as well as on-section enzyme histochemistry.

## Abstract (precis)

**Summary:** We propose a GMA embedding method that preserves fluorescent signals without the need for antibodies. The method complements whole-mount methods especially if advanced imaging technology is not readily available.

## INTRODUCTION

The increase in zebrafish genomic resources together with more sophisticated protocols for genome editing and other tools have contributed not only to unravel the genetic networks controlling development, but also generated zebrafish models with relevance to human disease (e.g. skeletal diseases, Laizé et al., 2014; Witten et al., 2017; Gistelinck et al., 2018). To assess the mutant phenotypes, many studies use fluorophores as marker molecules, whether genetically engineered in transgenic lines, in whole-mount *in situ* hybridization and fluorescent *in situ* staining, or as a fluorescent reporter in vital staining. Many of these studies, aiming at investigating the expression or function of disease-causing genes or localization of proteins, or perform cell tracking, rely on observations on whole-mount specimens. Obtaining cellular details, especially of structures located deep in the embryo, nevertheless offers a substantial added value to such studies. Furthermore, studies often focus on easily accessible or superficially exposed anatomical structures, such as caudal fin rays or scales for regeneration studies (e.g. Wehner and Weidinger, 2015; Cox et al., 2018), or the thin trunk of embryos for modeling vascular diseases (e.g. Hogan and Schulte-Merker, 2017). For a detailed phenotypic characterization, especially for internal structures, and/or structures that develop beyond the stage of complete transparency of the embryo, such as the skeletal system, it is helpful to complement whole-mount techniques and standard embedding and sectioning procedures, including paraffin or vibratome sections. Advanced imaging techniques such as dual photon microscopy or light sheet microscopy can overcome the limitations of observations on whole-mount embryos or superficially positioned structures. They also have the advantage of analysis in 3D, and enable *in vivo* analysis which can readily and precisely answer a broad range of biological questions, including those regarding dynamic cell movement (e.g. Liu et al., 2018). Yet, such imaging techniques require expensive equipment that is not readily available to most labs. Laser scanning confocal microscopy (LSCM) performed at whole-mount level can provide some information of internal structures but has limitations when deeper structures need to be imaged (Bruneel and Witten, 2015). Chemical clearing methods are under progress to allow deeper imaging (e.g. Watson et al., 2017), but even here, additional equipment is required. On the other hand, LSCM can be used to generate high-resolution pictures of immunostained sections obtained after paraffin embedding or cryotome sectioning (e.g. Schultz et al., 2018). However, fluorophores are not preserved in paraffin-embedded specimens, in contrast to cryosections. The downside of cryosections is the poor histological preservation, especially for heterogeneous tissues such as whole heads. Not only are anatomical structures in zebrafish small, cells are also smaller compared to mammalian cells. This is because cell size is tightly correlated to the total nuclear DNA content, expressed in picograms per cell nucleus. Compared to humans (3.50 pg DNA) zebrafish cells contain about half the amount of DNA per nucleus (1.80 pg DNA) while medaka cells contain only 1.09 pg DNA per nucleus. The small cell size requires higher precision in imaging and histological analyses (Witten et al., 2017). Thus, while standard histological techniques provide a convenient way to analyze tissues and cells, they need to be adapted for the use of fluorophores in zebrafish research.

In biological and biomedical research, different plastics (methacrylate or epoxy resins) are used in histology to obtain adequate cellular resolution. For light microscopical histology, glycol methacrylate (GMA, also termed HEMA, hydroxyethyl methacrylate) (Newman and Hobot, 2001) is widely acknowledged for its resolution, superior to paraffin. This embedding medium not only allows much thinner sections to be made, but also causes less distortion and superior cellular preservation in comparison to paraffin. GMA has a low viscosity and is therefore well suited for infiltration, and polymerization of the yolk-rich early embryos.

Commercially produced glycol methacrylate such as JB-4 or Technovit 7100 (Kulzer, Germany) has been shown to be suited to preserve EGFP (enhanced green fluorescent protein) labeling of transgenic whole embryos, and fluorescent signals after immunostaining (Sullivan-Brown et al., 2011). However, the array of fluorescent molecules currently used in zebrafish research has become vast, as is the range of applications in which they are used, including vital staining and enzyme histochemistry.

Here, we developed a new easy protocol for GMA embedding, serial sectioning and visualization of fluorescent signals in transgenic zebrafish, as well as in immunohistochemistry, cell tracking and enzyme histochemistry, with preservation of fluorescent signals at the tissue and cellular level. We use a non-commercial in-house prepared GMA (glycol methacrylate) plastic resin – a medium that results in excellent preservation of tissue morphology (Witten, 1997; Witten et al., 2001), as commercial GMA preparations present the disadvantage of not being available everywhere, and not allowing adjustment of its formulation in order to meet specific needs. GMA nevertheless has a drawback of not being electron-beam resistant and thus cannot be used for transmission electron microscopy (TEM) (Newman and Hobot, 2001). For correlative light and electron microscopy (CLEM) with preservation of fluorescence from genetically encoded fluorescent proteins, other acrylic resins are more suitable, such as Lowicryl (Nixon et al., 2009) or LR White (Bell et al., 2013). In return, GMA is more tolerant to water than either epoxy or polyester resins and does not require stringent conditions of dehydration. The whole procedure – from embryo preparation, embedding, sectioning and staining to visualization – can be accomplished in a few days. Overall, we demonstrate that GMA embedding can be part of daily routine, yielding cellular details with high resolution, thus nicely complementing methods that rely on observations of whole-mount embryos and providing a substitute if advanced imaging technology is not readily available.

## RESULTS AND DISCUSSION

We have previously proposed a method that allows high-resolution imaging on sections of whole-mount *in situ* hybridized embryos, using the epoxy resin Epon 812 (Verstraeten et al., 2012). However, embedding in epon does not preserve fluorescent signals, and thus another embedding medium is required. Glycol methacrylate suits this approach well. The use of a commercial version (JB-4) has been proposed before to reveal signals from ISH and whole-mount immunohistochemistry (Sullivan-Brown et al., 2011). Loizides et al. (2014) published a protocol for whole-mount staining of bone and cartilage followed by GMA embedding of the specimens for detailed histology. Here, we expand the number of applications that use fluorophores, including on-section methods, using a GMA made in-house.

### Embedding and visualization of transgenic zebrafish embryos

We established a simple protocol for visualization of GFP-positive or mCherry-positive cells after GMA embedding. Since 4% PFA is routinely used in most zebrafish labs, we employed this fixative as a standard. Fixed embryos can be kept in the fridge, but we noticed a loss of GFP signal strength over the course of weeks. Thus, embryos should be processed for embedding as rapidly as possible after fixation. Apart from a proper fixation, a critical step to avoid loss of fluorescent signal is dehydration. We tested graded series of both ethanol and acetone, varying the number and length of the steps. We obtained excellent preservation of embryos and fluorescence with a graded series of acetone (30, 50, 70, 80, 90, 100%), performed on ice. According to Xiong et al. (2014), quenched GFP molecules can be chemically reactivated to a fluorescent state by an alkaline buffer during imaging. We tested two alkaline buffers, Na_2_CO_3_ (0.1 M, pH 11.6) and NaOH (1 mM, pH 11), but concluded that they do not offer an advantage over the use of 1×PBS (pH 7.4). A drop of 1×PBS followed by coverslipping exposes the fluorescent signal well. It has been previously reported that application of water to (ultra-thin) sections collected from animals (in casu *C. elegans*) embedded in GMA immediately increased fluorescence intensity by 30%, and that this restoration of fluorescence suggests that a large fraction of the fluorescent proteins is maintained in a non-fluorescent, dehydrated state (Watanabe et al., 2011). The advantage of such a temporary mounting is that the coverslip can be flushed away, and the section reutilized for observation, provided it is dried at room temperature (RT) and stored in the dark. It can also be stained with DAPI even after long storage or the DAPI staining can be provided as a final step of IHC staining.

**Fig. 1.**
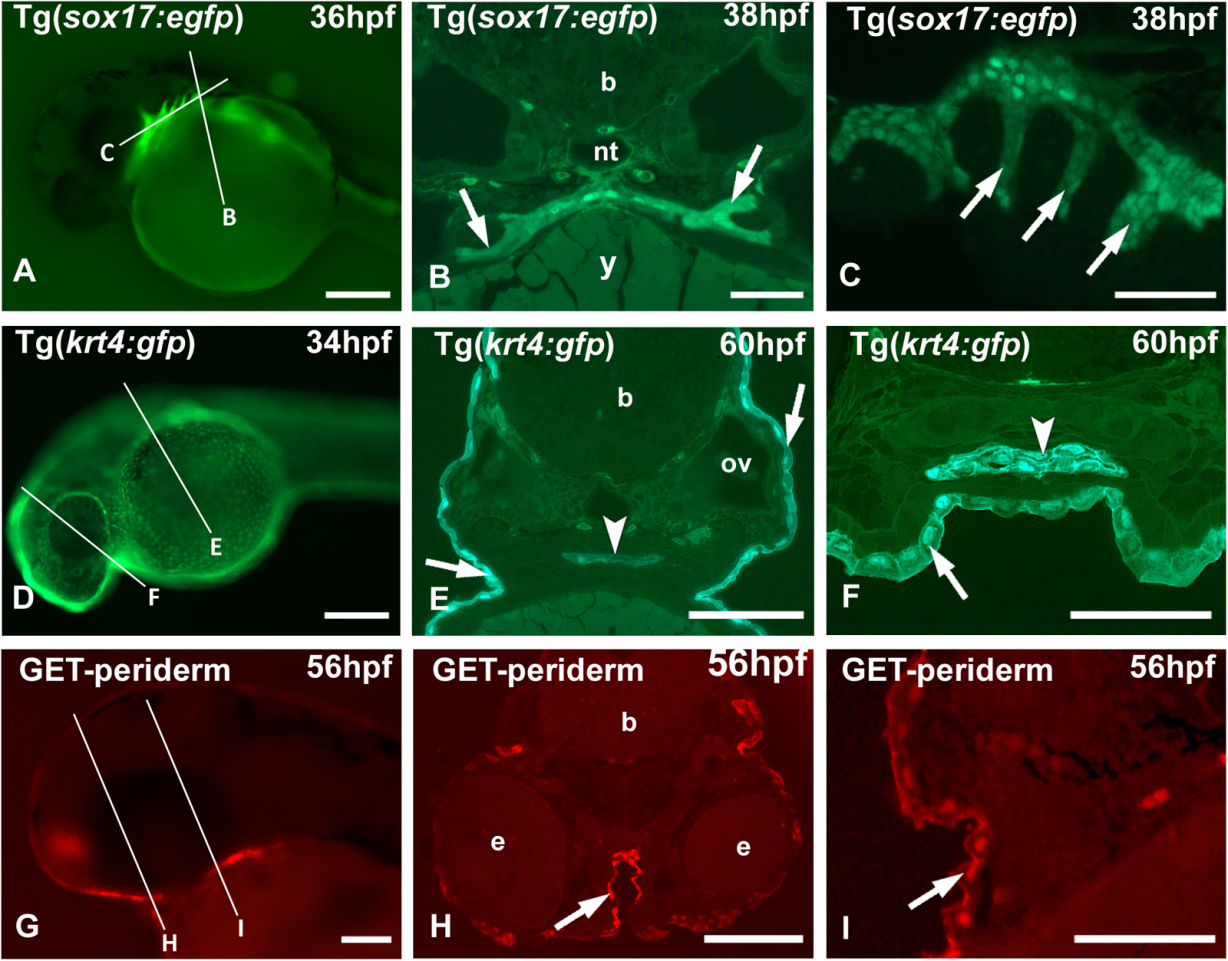
**Imaging of transgenic zebrafish lines.** (A,D,G) Whole-mount, (B,E,H) low- and (C,F,I) high-magnification (B,E,F,H,I) cross sections and (C) sagittal section. Lines in A,D,G indicate approximate level of sectioning in B,E,H and C,F,I. (A–C) Tg(*sox17:egfp*) zebrafish with GFP-positive endodermal cells along the midline, extending into the pouches (arrows). Blood vessels also show a fluorescent signal. (D–F) Tg(*krt4:gfp*) zebrafish with GFP-positive periderm (arrows). Labeled cells also cover the oropharyngeal lining (arrowheads). (G–I) Et(Gal4-VP16)^zc1044A^;Tg(UAS-E1b:nsfB-mCherry)^c264^, abbreviated as GET-periderm line (GET, Gal4 enhancer trap) (Eisenhoffer et al., 2017). The periderm is labeled red (arrows). b, brain; e, eye; nt, notochord; ov, otic vesicle; y, yolk. Scale bars: (A,D) 250 µm; (E,G,H) 100 µm; (B,C,F,I) 50 µm.

We show results of the protocol for three transgenic lines. In the transgenic Tg(*sox17:egfp*) zebrafish, *sox17* (SRY-Related HMG-Box Transcription Factor 17) drives GFP expression in the endoderm, including the paired endodermal pouches. Traditionally, the pouches are observed mostly on whole-mount embryos viewed from the side (Fig. 1A) (e.g. Crump et al., 2004; Lovely et al., 2016). On sections, the formation of the pouches can be easily monitored. Fig. 1B and C show a cross, resp. a sagittal, section of the pouches, clearly revealing a double layer of GFP-positive endodermal cells.

**Fig. 2.**
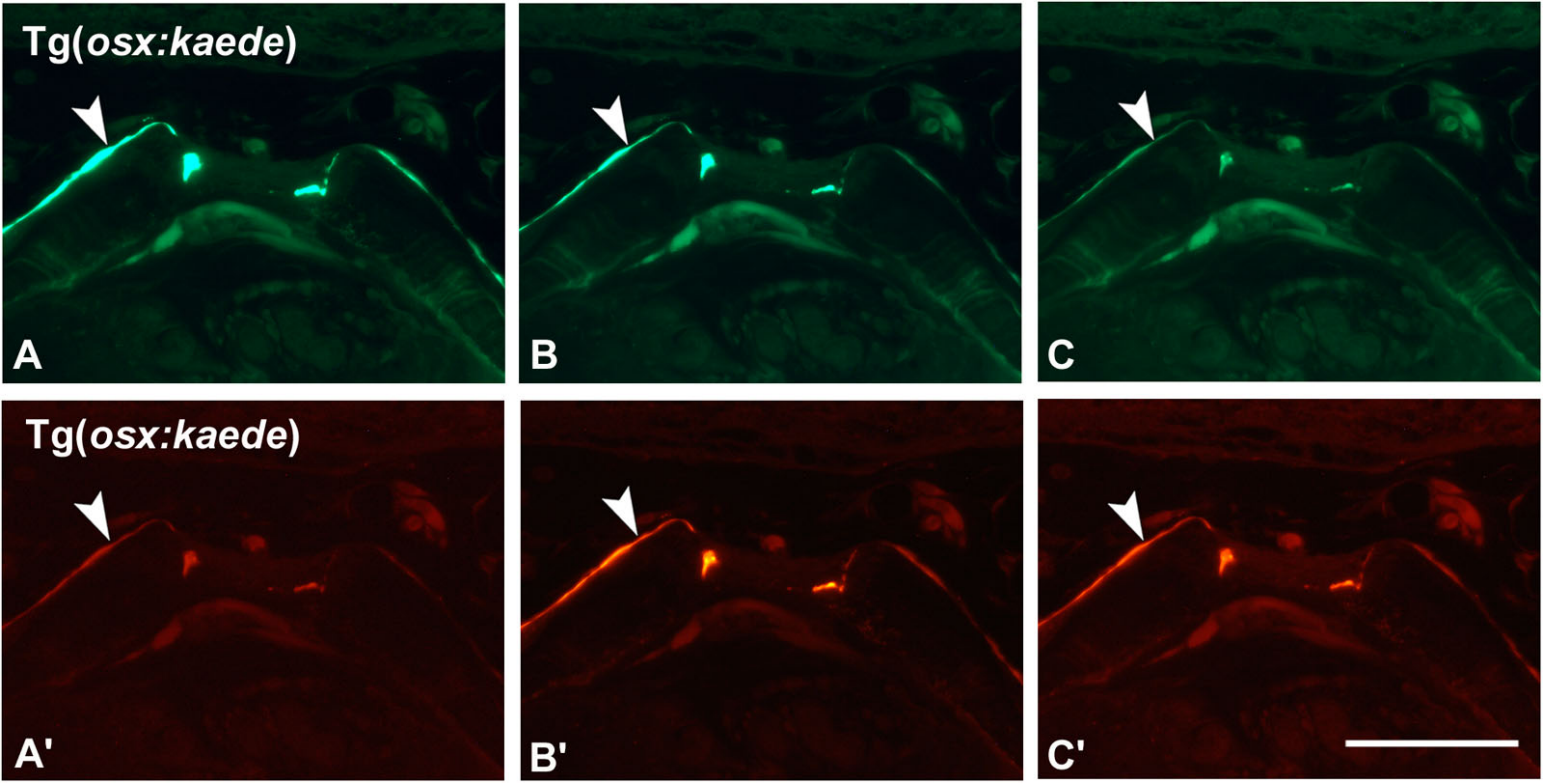
**Imaging of photoactivatable kaede.** Vertebral end plates of an adult Tg(*osx:kaede*) zebrafish shown using a GFP (A–C) and rhodamine (A′–C′) filter, before (A,A′), after 5 s (B,B′) and 30 s (C,C′) of exposure to a wavelength of 365 nm. Note the fading of the green signal from A to C and strengthening of the red signal from A′ to C′ in the osteoblasts lining the vertebral end plates (arrowheads). All pictures were taken using the same exposure time (1.6 s). Scale bar: 25 µm.

In zebrafish, the embryonic and larval epidermis is bi-layered, consisting of an outer layer, the periderm, deriving from the enveloping layer (EVL) (Kimmel et al., 1990; Fukazawa et al., 2010), and a basal keratinocyte layer (Le Guellec et al., 2004). This organization resembles the bi-layered organization of the mammalian epidermis at mid-gestation stages. The Tg(*krt4:gfp*) transgenic line uses promoter elements of the keratin 4 gene that drive expression of GFP confined to the periderm (Fig. 1D–F). On GMA sections, each GFP-positive cell of the periderm (Fig. 1F) can be clearly identified. The use of sections allows for imaging of labeled cells deep within the body, which would otherwise remain unnoticed when using whole-mount samples. Indeed, an advantage of the GMA sections used in the current manuscript is imaging deep structures not captured by confocal imaging or whole-mount images. For example, a layer of krt4-expressing cells covers the endodermal epithelium of the pharynx (Fig. 1E,F). Not just GFP but also other fluorescent proteins employed in transgenesis, such as mCherry, can be revealed in GMA sections using the protocol described above. For example, the recently developed transgenic lines to study the epidermis (Eisenhoffer et al., 2017; Fig. 1G) become even more powerful tools if examined via sections (Fig. 1H,I). Likewise, it is possible to reveal photoactivatable kaede (e.g. Cox et al., 2018) on sections, i.e. after dehydration and embedding (Fig. 2). This can be useful when working with double transgenics, e.g. in case only a GFP variant is available of a particular transgenic line. Another potential application is to rule out autofluorescence. While GMA formulations have been published that increase percentage of fluorescence preservation (e.g. Yang et al., 2013), the procedure described here is anticipated to maintain sufficient levels of fluorescence for most applications.

### Immunofluorescent localization of antibodies

The elucidation of spatiotemporal patterns of protein distribution in early embryos is a key prerequisite for understanding development. Again, many data obtained from immunostaining of embryonic zebrafish are presented on whole-mount embryos only, and an exact localization of the antibody is often lacking, especially for deeper lying structures.

**Fig. 3.**
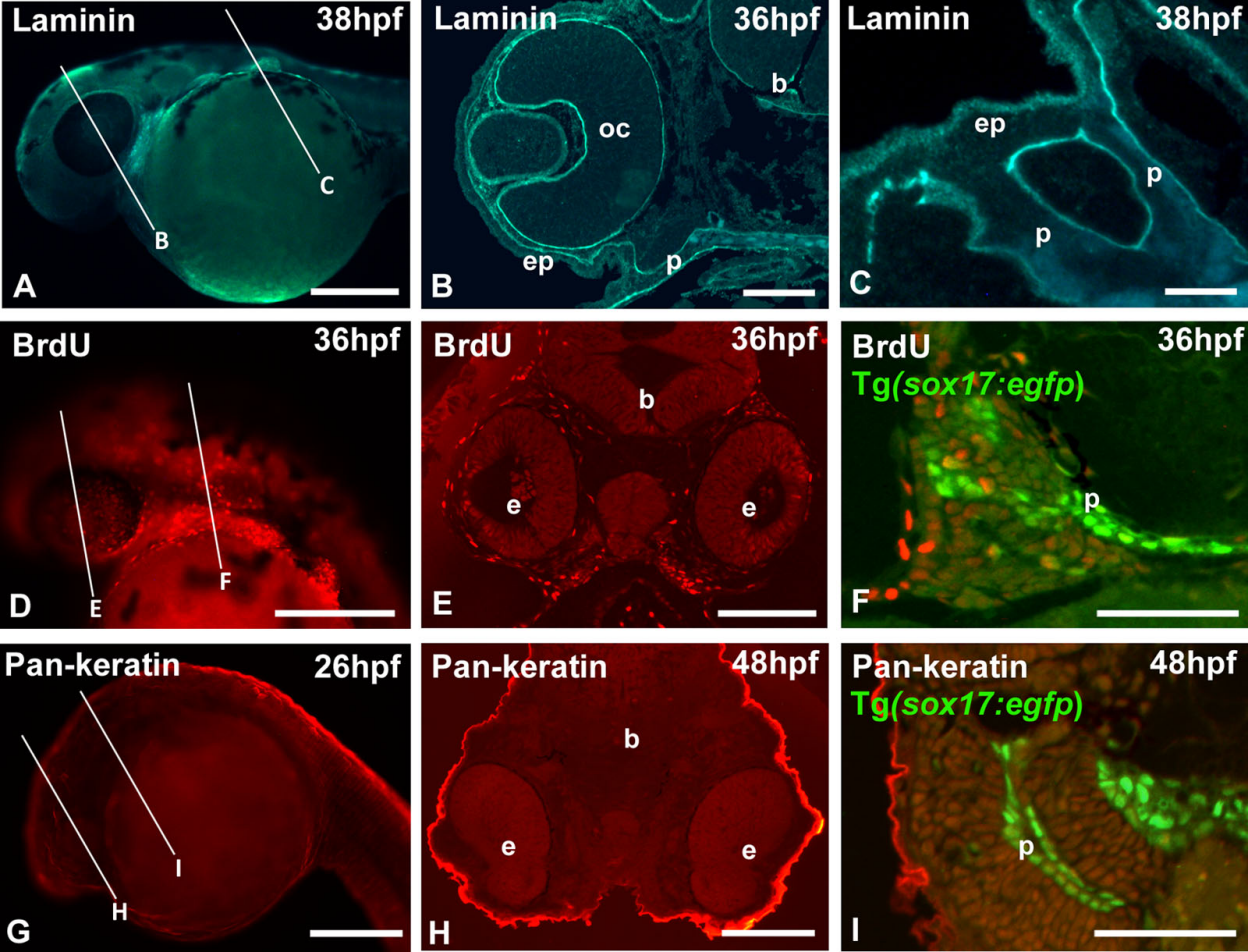
**Whole-mount immunostaining.** (A,D,G) Whole-mount, (B,E,H) low- and (C,F,I) high-magnification cross sections. Lines in A,D,G indicate approximate level of sectioning in B,E,H and C,F,I. (A–C) Wild-type (WT) zebrafish immunostained for laminin. Note the basal lamina delimiting optic cup, brain, epidermis and endodermal pouches. (D–F) Tg(*sox17:egfp*) zebrafish pulse-labeled with BrdU and whole-mount stained with an anti-BrdU antibody. (G-I) Tg(*sox17:egfp*) zebrafish immunostained with a pan-cytokeratin antibody. The whole-mount images in D,G show the red channel only; the GFP fluorescence is likewise visible prior to dehydration and embedding. b, brain; e, eye; ep, epidermis; oc, optic cup; p, endodermal pouch. Scale bars: (A,D,G) 250 µm; (E,H) 100 µm; (B,F,I) 50 µm; (C) 25 µm.

We routinely use standard procedures for whole-mount immunohistochemistry (IHC) followed by embedding the samples in GMA. As indicated before (Sullivan-Brown et al., 2011), immunofluorescent techniques work on whole-mount embryos only, not on GMA sections. For immunofluorescent localization of antigens on plastic sections, it is recommended to use other acrylic resins such as LR White (Newman and Hobot, 2001; Luby-Phelps et al., 2003) or methyl methacrylate – that has nevertheless to be removed from the sections prior to staining (Hand and Blythe, 2016; and references therein). Fig. 3A–C present the results of whole-mount anti-laminin immunofluorescent staining followed by GMA embedding and processing as described above, to visualize the basement membranes. Laminins are large glycoprotein heterotrimers that are found as major components of basement membranes in almost every animal tissue (Colognato and Yurchenco, 2000; Ekblom et al., 2003). Basement membranes play an important role in tissue development and maintenance including mechanical stability, promotion of cell adhesion, migration, growth and differentiation. Laminin protein, and by extension basement membranes, are clearly identifiable below the epidermis, around the brain and in ocular structures, particularly in the developing and mature lens (cf. Hallmann et al., 2005) (Fig. 3B). Anti-laminin also serves as a convenient marker to show boundaries between epithelial and mesenchymal tissues in sections (Fig. 3C).

Cell proliferation is an essential process during growth and development and often examined using BrdU. We pulse-labeled 36 hpf embryos and used a standard procedure to reveal BrdU on whole-mount embryos (Fig. 3D). After embedding and sectioning, the distribution of labeled cells can be precisely mapped and if necessary quantified (Fig. 3E). BrdU can also be administered to transgenic embryos and the fluorescent secondary antibody can be revealed simultaneously with the fluorophore used in the transgene (Fig. 3F).

In another example combining different fluorophores, we used a pan-cytokeratin antibody on Tg(*sox17:egfp*) embryos (Fig. 3G–I). The latter two examples show that the fluorescence of GFP (as well as of other fluorophores) is maintained through the whole-mount immunolabeling procedure as well as the GMA embedding protocol. The GFP can also be visualized in the whole-mount embryo prior to embedding. While immunolabeling of transgenic GFP-labeled embryos is a common procedure, the resolution obtained by processing whole embryos into sections is a clear asset.

### Visualization of fluorescent dyes used for cell tracking

**Fig. 4.**
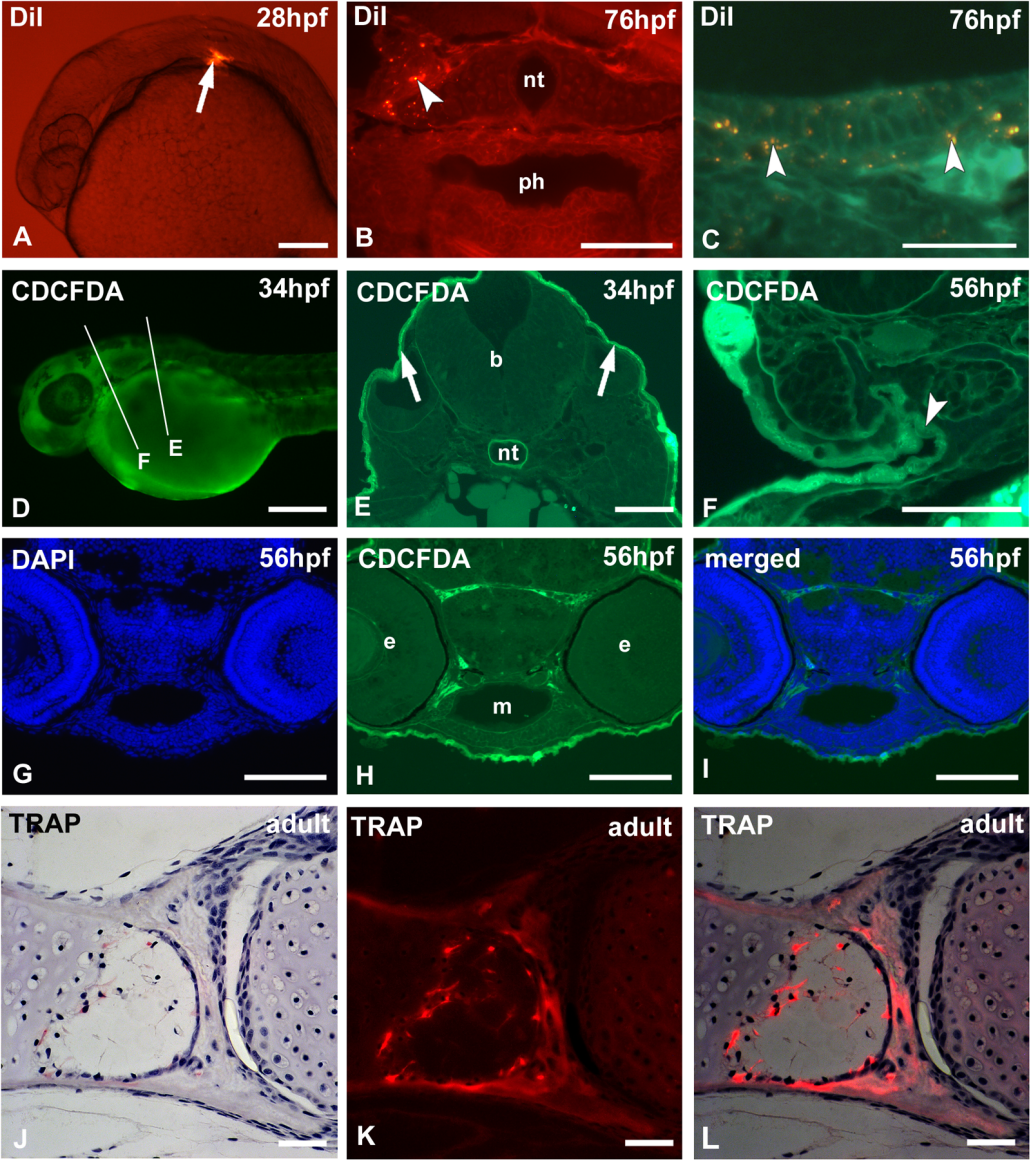
**Cell tracking with fluorescent vital dyes and on-section histochemistry.** (A–I) Cell tracking. (A,D) Whole-mount, (B,E) low- and (C,F) high-magnification cross sections. (A–C) WT zebrafish injected with DiI (A, arrow) at 28 hpf and euthanized after 48 h reveal areas of DiI distribution (arrowheads); the DiI label (yellowish dots) stands out sharply against an autofluorescent background (B,C). (D–F) WT zebrafish vital stained with CDCFDA. Only cells exposed to the solution (periderm) take up the stain (E, arrows). After a chase time of 22 h, labeled cells can be observed inside the forming gill slit (F, arrowhead). Lines in D indicate approximate level of sectioning in E,F. (G–I) DAPI staining (G) on section of CDCFDA labeled embryo (H) and merged picture (I). (J–K) On-section histochemistry for the osteoclast marker TRAP, showing the palatoquadrate and palatine bone in adult WT zebrafish, viewed in transmitted light (J), under epifluorescence (K) and the overlay of transmitted light and epifluorecence (L). b, brain; e, eye; m, mouth; nt, notochord; ph, pharyngeal lumen. Scale bars: (A,G,H,I) 100 µm; (D) 250 µm; (B,E,F) 50 µm; (C,J,K,L) 25 µm.

Lineage tracing and cell fate determination relies for a large part on the use of fluorescent cell tracers. DiI is a lipophilic dye that is usually administered via injection (Fig. 4A). It is weakly fluorescent until incorporated into membranes and is used as long-term tracer for neuronal and other cells. DiI can be revealed on sections after ethanol dehydration and embedding in GMA (Fig. 4B,C).

We also used vital staining with CDCFDA to label the external surface of the embryo (the periderm) and to establish the fate of these cells (Fig. 4D). CDCFDA is a non-fluorescent molecule that diffuses into cells and is hydrolyzed by intracellular non-specific esterases to give a fluorescent product (similar to CCFSE, used by Shone and Graham, 2014). The fluorescent product accumulates only in those cells that have intact cell membranes (Griffith and Hay, 1992); therefore, dead cells with leaky membranes are not stained. Fig. 4E and F show how labeled peridermal cells invade the embryo via the gill slits.

### On-section staining

DAPI is a widespread nuclear counterstain that is often applied on whole-mount embryos. DAPI can also easily be applied on sections (Fig. 4G–I). The result is comparable to whole-mount staining followed by embedding.

Various enzymes that are involved in skeletal modeling and remodeling can be studied after GMA embedding and on-section immunohistochemistry in order to map their precise spatial and temporal pattern of activity. For example, tartrate-resistant acid phosphatase specifically marks cells responsible for bone resorption (osteoclasts) (Witten et al., 2001; Witten and Huysseune, 2009). In zebrafish, cells expressing TRAP are small in size and mononuclear, at least in early development, and therefore not easily revealed on conventionally stained sections. We have successfully employed GMA embedding and TRAP staining on sections of both larval and adult zebrafish (Witten et al., 2001; Kague et al., 2018). The reaction product can be visualized both in bright field (Fig. 4J) and under epifluorescence (Fig. 4K), and images can be overlain (Fig. 4L). Other enzymes can be revealed after GMA embedding such as ATPase, alkaline phosphatase or alpha naphtyl acetate esterase (Witten, 1997; Witten et al., 1999; Witten et al., 2000; Witten et al., 2001).

### Conclusion

In conclusion, we propose an easy-to-perform GMA embedding method that preserves fluorescent signals and allows visualization of GFP, mCherry and other fluorophores on semithin sections (3 µm or less) without the need for antibodies. The method is suited for a wide range of applications including study of transgenic zebrafish of unlimited size, immunostaining of whole-mount embryos, cell tracking and on-section enzyme histochemistry of embryonic or even adult zebrafish. In addition, tissue preservation is superior to any of the other common procedures used in histology (such as paraffin and vibratome sections) and allows a detailed study of cells in their tissue or organ context. The method nicely complements whole-mount methods especially if advanced imaging technology is not readily available.

## MATERIALS AND METHODS

### Ethical statement

Animal care, experimentation and euthanasia complied with European Directive 2010/63/EU of 22 September, 2010. The experimental protocol and all animal procedures used in this study were approved by Ghent University (laboratory permit number LA1400452).

### Zebrafish lines and collection of transgenic fish

Wild-type AB line and transgenic Tg(*sox17:egfp*) zebrafish (*Danio rerio*) lines (Mizoguchi et al., 2008) were obtained from the laboratory of Dr R. Opitz (VUB, Brussels, Belgium). Tg(*krt4:gfp*) (Gong et al., 2002) were a gift from the laboratory of Dr M. Hammerschmidt (University of Köln, Köln, Germany). Et(Gal4-VP16)^zc1044A^;Tg(UAS-E1b:nsfB-mCherry)^c264^, abbreviated as GET-periderm line (GET, Gal4 enhancer trap) were obtained from the laboratory of Dr G. Eisenhoffer (MD Anderson Cancer Center, Houston, Texas, USA) (Eisenhoffer et al., 2017). An unpublished Tg(*osx:kaede*) strain was made available by Dr Kenneth D. Poss and Sumeet P. Singh (Duke University, Durham, NC, USA). All zebrafish were maintained and bred in accordance to Westerfield (1993). WT and transgenic embryos were kept at 28.5°C until euthanasia by an overdose of 1% MS222 (ethyl 3-aminobenzoate methane sulfonate, cat No.: E10521, Sigma Aldrich). They were staged according to Kimmel et al. (1995), fixed in 4% paraformaldehyde (PFA) in PBS buffer (pH 7.2) for 2 h at RT, and either processed immediately for embedding or stored in methanol at −20°C until immunostaining. Adult transgenic zebrafish were fixed overnight at 4°C in 4% PFA in PBS buffer (pH 7.2), rinsed in tap water for 1 h, and decalcified with 10% EDTA in Tris buffer (100 mmol, pH 7.2) for 48 h.

### Immunofluorescence

We used whole-mount immunofluorescent staining according to established protocols to detect various antigens (laminin, pan-cytokeratin, BrdU). In all cases, WT and transgenic embryos were euthanized by an overdose of 1% MS222, fixed in 4% PFA for 10 min at RT, and then overnight at 4°C (anti-laminin staining), or just for 2 h (for anti-BrdU and anti-pan-cytokeratin staining) followed by storage in 100% methanol at −20°C.

The protocol for whole-mount immunostaining for laminin follows O'Brien et al. (2011), with adjustment for the time of permeabilization. Following fixation, embryos were permeabilized in acetone for 10 min at –20°C (the time for permeabilization depends on the developmental stage of the embryos, see Table 1). The embryos were rinsed in 1×PBS (phosphate-buffered saline) (pH 7.2) and the primary antibody (anti-α-laminin, 1:100, cat. no.: L9393, Sigma Aldrich) was applied overnight at 4°C. On the following day, embryos were incubated in the secondary antibody (goat anti-rabbit DyLight 488 nm, 1:200, Abcam) for 4 h at RT. Embryos were rinsed in 1×PBS. After antibody staining, embryos were rinsed in 1×PBS again and stored in 4% PFA until processing for GMA embedding.

**Table 1.**
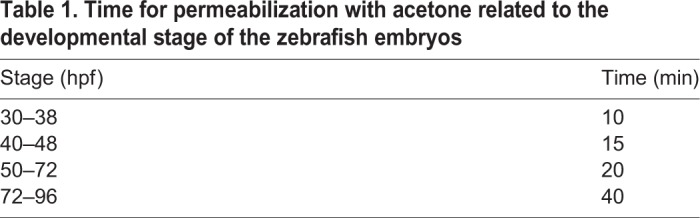
**Time for permeabilization with acetone related to the developmental stage of the zebrafish embryos**

For proliferation studies, 36 hpf embryos were pulse-labeled for 20 min in 10 mM BrdU (5-bromo-2′-deoxyuridine)/15% dimethylsulfoxide and sacrificed immediately after BrdU administration, following the protocol described in Verduzco and Amatruda (2013). Whole-mount immunostaining for BrdU was performed following this same protocol, using an anti-BrdU (mouse, 1:100, cat. no.: B2531-2ML, Sigma Aldrich) as primary antibody, and an Alexa Fluor^®^ 594 (goat anti-mouse IgG, 1:200, Abcam) as secondary antibody. Embryos were next rinsed and kept in 4% PFA until processing for GMA embedding.

The same protocol (Verduzco and Amatruda, 2013, from step 4 onwards) was employed for whole-mount immunostaining of keratins using a pan-cytokeratin (AE1/AE3) (mouse, Santa Cruz, 1:200) as primary antibody, and an Alexa Fluor^®^ 594 (goat anti-mouse IgG, 1:200, Abcam) as secondary antibody.

Negative controls were performed by omitting the primary antibody from the reaction mixture.

### Cell tracking

Two molecules for cell tracking were used: DiI and CDCFDA. DiI is a lipophilic dye commonly used for cell fate tracing. A stock solution of 5 µg/µl DiI (Invitrogen Cell Tracker CM-DiI, cat no. C-7000) was prepared by diluting 50 µg in Ethanol 1:10, and stored at −20°C. A dilution of 1:10 in 0.3 M Sucrose (made up in nuclease-free water from Sigma Aldrich) was used to inject dechorionated WT embryos of 28 hpf. For injection, ca. 5 dechorionated embryos were placed on a smooth agar plate using a plastic balloon pipette. All excess water was sucked up with a plastic balloon pipette and one drop of MS222 0.001% was sprinkled on the embryos. Excess fluid was sucked away. Embryos were euthanized at appropriate times using an overdose of 1% MS222, fixed in 4% PFA for 24 h, and transferred to 1×PBS with 0.02% sodium azide for storage at 4°C, until embedding.

CDCFDA [5-(and-6)-carboxy-2′,7′-dichlorofluorescein diacetate, succinimidyl ester, mixed isomers, cat. no.: 22026, AAT Bioquest, Inc.] is an isomer mixture of CCFSE–[5-(and-6)-carboxy-2′,7′-dichlorofluorescein diacetate, succinimidyl ester] used by Shone and Graham (2014). It was dissolved in anhydrous DMSO (dimethylsulfoxide) to 50 mM and stored at −20°C. A working concentration (20, 100, 250, 500 µM) in 1×PBS was used. WT embryos varying in age between 8 and 22 hpf, as well as some older embryos (30, 40 and 56 hpf) were soaked in CDCFDA for 4 h, rinsed in egg water and transferred to fresh egg water. At selected time points, embryos were euthanized by an overdose of 1% MS222, fixed in 4% PFA and stored in 4% PFA until processing for GMA embedding.

### GMA embedding and sectioning

We used a GMA embedding protocol as described by Witten et al. (2001). Briefly, the zebrafish were rinsed in two changes of 1×PBS (15 min each) and dehydrated using a graded series of acetone solutions [30, 50, 70, 80, 90, 100% for 15 min each (embryos) or 30, 60, 100% for 1 h each (adults)]. These steps were performed on ice, sheltered from light, using a shaker. Embryos were then impregnated in several changes of fresh glycol methacrylate monomer solution for 15 min at 4°C (two changes), followed by 60 min at 4°C. Adults were kept in the monomer for several days. Glycol methacrylate monomer solution consists of 80 ml (2-hydroxyethyl)-methacrylate (+200 ppm p-methoxyphenol, usually already in the product, cat. no.: 17348-250ML-F, Sigma Aldrich), 12 ml ethylene glycol monobuthyl ether (cat. no.: 537551-1L.A, Sigma Aldrich) and 270 mg benzoyl peroxide (added to the solution and stirred overnight). The samples were subsequently transferred to fresh GMA monomer solution and left for 24 h at 4°C. After a total of 24 h in the monomer, the samples were placed in an embedding mold (PTFE Flat embedding mold, Electron Microscopy Sciences) containing GMA with 2% catalyst (N,N-dimethylaniline 1 ml+poly-ethylene glycol-200 10 ml, Sigma Aldrich) added. A slice of polymerized GMA was placed at the bottom of each well prior to positioning the fish in order to prevent the sample from sinking to the bottom of the well (hence side of the block). Care was taken during polymerization to protect the blocks from air by covering the embedding mold with an oxygen barrier film trimmed to the appropriate size (ACLAR 33C embedding film). Polymerization occurred, sheltered from light, at 4°C for 24 h (embedding mold placed on crushed ice) and another 24 h at RT. The GMA block was removed from the mold and stored at RT in the dark. For sectioning, the block was mounted on a standard histology microtome (Microm HM360, Prosan) and routinely sectioned at 3 µm (down to 1 µm) using disposable knives (Technovit Histoblade knives, Kulzer). Sections were placed on a drop of demineralized water on a glass slide and air-dried at RT, sheltered from light.

### On-section enzyme histochemistry

For TRAP staining, zebrafish were euthanized with an overdose of MS222, fixed in cold 100% acetone and stored at −20°C prior to dehydration and embedding, as long-term storage in PFA destroys enzyme activity. TRAP staining was carried out on sections of GMA embedded tissues, using naphthol AS-TR phosphate (N-AS-TR-P, cat. no.: N6125-1G, Sigma Aldrich) as substrate and hexazotized pararosaniline (PRS, Acros Organics 227881000, Thermo Fisher Scientific) as color component, following the protocol of Witten et al. (2001). After final rinsing with demineralized water, sections were air-dried. Controls are performed either by (a) heating the section at 90°C for 10 min prior to incubation, (b) incubation without substrate, (c) incubation without tartrate or (d) adding NaF (10 mmol/l) to the incubation solution (Witten, 1997).

### Nuclear counterstaining

When required, a nuclear counterstain was applied either before or after imaging the sections using DAPI (4′, 6-diamidino-2-phenylindole, dihydrochloride, cat. no.: D9542-1MG, Sigma Aldrich). To this end, sections were covered with a drop of DAPI (1:1000 in 1×PBS) for 15 min at RT, sheltered from light, and washed three times for 10 min with 1×PBS. Other methods of counterstaining, such as with Toluidine Blue, are necessarily performed after imaging since these techniques destroy the fluorescent signal.

### Imaging

Fluorescent signals were visualized by covering the sections with a drop of 1×PBS, followed by coverslipping. Sections were observed with a Zeiss Axioimager Z1, equipped for epifluorescence, using the following filters: GFP (Excitation BP 470/40, Beam splitter FT 495, Emission BP 525/50), Rhodamine (Excitation BP 546/12, Beam splitter FT 560, Emission BP 575-640) and DAPI (Excitation G 365, Beam splitter FT 395, Emission BP 445/50), and photographed with a Zeiss Axiocam 503 camera using ZEN software (www.zeiss.com). After imaging, the coverslip was removed by flushing with demineralized water, and the section was allowed to dry for renewed observation or for counterstaining and permanent mounting.
